# Prevalence of Scabies and Impetigo 3 Years After Mass Drug Administration With Ivermectin and Azithromycin

**DOI:** 10.1093/cid/ciz444

**Published:** 2019-05-25

**Authors:** Michael Marks, Lucia Romani, Oliver Sokana, Lazarus Neko, Relmah Harrington, Titus Nasi, Handan Wand, Margot J Whitfeld, Daniel Engelman, Anthony W Solomon, John M Kaldor, Andrew C Steer

**Affiliations:** 1 Clinical Research Department, Faculty of Infectious & Tropical Diseases, London School of Hygiene & Tropical Medicine, London, United Kingdom; 2 Hospital for Tropical Diseases, London, United Kingdom; 3 Kirby Institute, University of New South Wales, Sydney; 4 Murdoch Children’s Research Institute, Melbourne, Australia; 5 Ministry of Health and Medical Services, Honiara; 6 Ministry of Health and Medical Services, Choiseul; 7 Atoifi Health Research Group, Malaita, Solomon Islands; 8 St Vincent’s Hospital, University of New South Wales, Sydney; 9 Centre for International Child Health, University of Melbourne, Melbourne, Australia

**Keywords:** ivermectin, scabies, impetigo, mass drug administration, neglected tropical diseases

## Abstract

**Background:**

Ivermectin-based mass drug administration has emerged as a promising strategy for the control of scabies and impetigo in settings where the diseases are endemic. Current follow-up data are limited to 12 months for the majority of studies. Longer-term data are vital to inform the sustainability of interventions.

**Methods:**

We conducted a prevalence survey for scabies and impetigo in 10 villages in Choiseul Province of the Solomon Islands 36 months after a single round of ivermectin and azithromycin mass drug coadministration. In the primary analysis, we compared the prevalence of scabies and impetigo at 36 months to the prevalence at baseline.

**Results:**

At 36 months, the prevalence of scabies was 4.7% (95% confidence interval [CI], 3.6–6.1), which was significantly lower than at baseline (18.7%; relative reduction, 74.9%; 95% CI, 61.5%–87.7%; *P* < .001). The prevalence of impetigo was 9.6% (95% CI, 8.1%–11.4%), significantly lower than at baseline (24.7%; relative reduction, 61.3%; 95% CI, 38.7%–100%; *P* < .001). The highest prevalence of scabies was among children aged <5 years (12.5%; adjusted odds ratio, 33.2; 95% CI, 6.6–603.2), and the highest prevalence of impetigo was among children aged 5–9 years (16.4%; adjusted odds ratio, 8.1; 95% CI, 3.6–21.8).

**Conclusions:**

There was a sustained impact of a single round of ivermectin and azithromycin mass drug coadministration on the prevalence of scabies and impetigo 3 years after the intervention. Our data provide further support to adopt this intervention as a central component of global scabies control efforts.

**Clinical Trials Registration:**

Australian and New Zealand Trials Registry (ACTRN12615001199505).

## Teaser

Ivermectin-based mass drug administration is a promising strategy for scabies. Most studies only report 12-month outcomes. We demonstrate that the prevalence of scabies and impetigo remain low 3 years after large-scale intervention in the Solomon Islands.

Scabies, caused by infestation with the mite *Sarcoptes scabei var hominis*, is a significant public health problem in many tropical countries and is particularly common in the Pacific region [1–3]. In these settings, the population prevalence of scabies can be as high as 20%, with peak prevalence as high as 50% among children [2]. Infestation with scabies results in inflammation and itch and is frequently associated with secondary bacterial infection (impetigo) caused by *Staphylococcus aureus* and *Streptococcus pyogenes* (impetigo) [1, 4]. These infections can result in more serious complications including septicemia, poststreptococcal glomerulonephritis, acute rheumatic fever, and rheumatic heart disease [4].

In highly endemic settings, the treatment of individuals and their immediate contacts has no impact on the overall community prevalence of scabies due to high rates of reinfestation [5, 6]. Because of this, there is increasing interest in population-level interventions for the control of scabies. Both permethrin- and ivermectin-based mass drug administrations (MDAs) have demonstrated efficacy in single-arm studies [7–10]. More recently, ivermectin-based MDA was shown to be superior to alternative strategies, including permethrin MDA and standard care, in a comparative trial [11]. A consistent finding in these studies is a substantial reduction in impetigo of 60%–90% following treatment of scabies [7, 11, 12].

There are limited data on the time course with which prevalence rebounds following intervention. Most recent published data have focused on 12-month outcomes following a single round of MDA [11–13]. In an early study that used permethrin-based MDA, scabies prevalence rebounded within 3 months of cessation of the intervention [7], but minimal prevalence rebound has been reported at 1 year after ivermectin-based MDA [11, 14]. Understanding the longevity of the intervention effect is crucial for planning public health measures for scabies, including the timing for additional rounds of MDA. Only a single study has reported long-term outcomes in the context of multiple rounds of intervention [14], but there are no data on long-term outcomes following a single round of MDA.

In 2015, we conducted a study to assess the prevalence of scabies and impetigo before and after an ivermectin-based MDA in a population of more than 25 000 individuals in the Solomon Islands. We demonstrated a substantial reduction in the prevalence of both scabies and impetigo at 12 months [13, 15]. To assess the durability of reductions in prevalence following MDA and inform decisions about the optimal interval between rounds of MDA, we conducted a follow-up evaluation 36 months after the intervention.

## METHODS

The methods of the Azithromycin Ivermectin MDA (AIM) study have been reported in detail elsewhere [13, 15]. Briefly, AIM was a single-arm, before-and-after trial conducted in Choiseul Province, 1 of 10 administrative provinces of the Solomon Islands. The population was estimated at 26 372 in 2009, with the majority of residents on a single large island. The study made use of the infrastructure of a planned azithromycin MDA conducted for the elimination of trachoma as a public health problem [16]. In collaboration with the Ministry of Health and Medical Services (MHMS), we integrated an ivermectin-based MDA for the control of scabies into this routine activity. At baseline (2015), all residents in Choiseul Province were offered treatment for both trachoma and scabies. Treatment for trachoma consisted of a single dose of oral azithromycin or (when azithromycin was contraindicated) a self-administered 6-week course of 1% topical tetracycline eye ointment. Treatment for scabies consisted of 2 doses of oral ivermectin (200 µg/kg) or (when ivermectin was contraindicated, specifically, for pregnant or breast-feeding women and children aged <5 years) topical permethrin. Participants who required permethrin were given the option to have a trained nurse apply the cream in a private room or to apply it themselves at home. The first dose of treatment for scabies was administered at the time of treatment for trachoma, and the second dose was offered 7 to 14 days later. At baseline and at 12 months, 10 villages were randomly selected, and all individuals were offered enrollment and examination for scabies and impetigo. We have previously reported both safety outcomes and the 12-month efficacy results with regards to scabies and impetigo [13, 15]. Overall, AIM enrolled 26 188 participants who, based on the 2009 census, represented 99.3% of the population. The estimated population coverage of the initial dose of treatment for scabies was 98.6%; that of the second dose of treatment for scabies was 83.7%.

For the 36-month evaluation, we utilized the same survey methodology as at baseline and 12 months. We obtained a complete list of villages in Choiseul Province with estimated populations from the MHMS. We used simple random selection to identify 10 villages with populations of 100–250 residents to participate in our 36-month survey. Villages selected in either (or both) the baseline and 12-month survey were eligible for inclusion in the 36-month survey.

All residents of the selected villages were eligible to participate in the survey. We obtained written informed consent from adults and from the parents/guardians of children. All individuals were examined by a clinician experienced in the diagnosis of scabies and impetigo (M. M.). We used the same clinical diagnostic methods that were used in the baseline and 12-month surveys. Scabies was defined as pruritic inflammatory papules with a typical anatomical distribution, such as the webs of the fingers, hands, wrists, and ankles, as per Integrated Management of Childhood Illness guidelines [17, 18]. Examination excluded breasts and genitals, unless requested by participants and then only in a separate, private space. Impetigo was defined as pustular or ulcerative lesions surrounded by erythema. All data were collected directly into the OpenDataKit package on Android devices.

### Statistical Analyses

For the primary analysis, we compared the prevalence across survey villages of scabies and impetigo at 36 months to the prevalence at baseline. We calculated the absolute (difference between baseline and month 36) and relative reductions in prevalence and their 95% confidence intervals (CIs) based on their respective variances [19]. We compared prevalence using a χ ^2^ test and considered *P* < .05 to be statistically significant.

In further secondary analysis, we fitted a multilevel logistic regression model to calculate the odds of scabies and impetigo at 1 and 3 years of follow-up compared to baseline after controlling for age and gender. Then, we adjusted for clustering by including a random effect for study village. We calculated the population-attributable fraction of impetigo due to scabies at baseline, 12 months, and 36 months. We calculated the absolute change in prevalence of both scabies and impetigo between our 12-month and 36-month surveys. Finally, we performed a subgroup analysis on the subset of villages visited more than once. All analyses were conducted in R version 3.5.1 [20].

### Registration and Ethical Approval

The trial was registered with the Australian and New Zealand Trials Registry (ACTRN12615001199505) and approved by the Solomon Islands National Research Ethics Committee (15/33) and the Royal Children’s Hospital Human Research Ethics Committee (35148A). Ethics amendments were granted to undertake the 36-month follow-up survey. As previously reported, an independent data safety monitoring committee oversaw the original trial intervention.

## RESULTS

At the 36-month survey, we examined 1210 individuals across the 10 selected villages, which represented 83.3% of the resident population of those villages. (At the baseline and 12-month surveys, we had examined 1399 and 1261 individuals, respectively, representing 84.2% and 77.6% of the village populations [13].) The demographics of enrolled participants were similar at each survey (Table 1). The median age of participants in the 36-month survey was 12 years (interquartile range, 8–32), and 546 (45.1%) were male. Four of the selected villages had been surveyed at baseline; 2 selected villages had been surveyed at the 12-month survey.

**Table 1.** Demographic Characteristics of Participants in Each Survey

Characteristic		Baseline Survey	12-Month Survey	36-Month Survey
Gender, n (%)	Female	711 (50.8)	691 (54.8)	664 (54.9)
	Male	688 (49.2)	570 (45.2)	546 (45.1)
Age, mean (standard deviation), y		21.6 (18.6)	22.2 (18.6)	20.3 (18.5)
Age group, n (%), y	<5	231 (16.5)	155 (12.3)	160 (13.2)
	5–9	250 (17.9)	229 (18.2)	269 (22.2)
	10–14	225 (16.1)	209 (16.6)	277 (22.9)
	15–24	175 (12.5)	227 (18.0)	121 (10)
	25–34	177 (12.7)	130 (10.3)	118 (9.8)
	≥35	341 (24.4)	311 (24.7)	265 (21.9)

### Prevalence in Sentinel Villages at Baseline and 36 Months

At the 36-month survey, 57 individuals were diagnosed with scabies (4.7%; 95% CI, 3.6–6.1). The prevalence of scabies at 36 months was significantly lower than at baseline (18.7%), with an absolute reduction of 14.0% and relative reduction of 74.9% (95% CI, 61.5–87.7%; *P* < .001; Table 2). The prevalence of scabies varied across the 10 villages from 0.0% to 18.7% (Supplementary Table 1).

**Table 2.** Prevalence of Scabies and Impetigo at Baseline, 12, and 36 Months

	Prevalence at Baseline(95% CI)n/N	Prevalence at 12 Months(95% CI) n/N	Prevalence at 36 Months(95% CI)n/N	Absolute Reduction in Prevalence at 12 Months^a^(95% CI)	Relative Reduction in Prevalence at 12 Months^a^(95% CI)	Absolute Reduction in Prevalence at 36 Months^a^(95% CI)	Relative Reduction in Prevalence at 36 Months^a^(95% CI)	Absolute Change in Prevalence Between 12 and 36 Months(95% CI)
Scabies	18.7% (16.6 to 20.8) (261/1399)	2.3% (1.6 to 3.3) (29/1261)	4.7% (3.6 to 6.1) (57/1210)	16.6% (14.5 to 18.8)	89% (77.5 to 100)	14.0% (11.5 to 16.4)	74.9% (61.5 to 87.7)	+2.4% (+0.1 to + 3.9)
Impetigo	24.8% (22.6 to 27.2) (347/1399)	6.4% (5.2 to 8.0) (81/1261)	9.6% (8.1 to 11.4) (116/1210)	18.7% (16.2 to 21.3)	75% (65.2 to 85.7)	15.2% (9.6 to 24.8)	61.3% (38.7 to 100)	+3.2% (+0.9 to +6.4)

Abbreviation: CI, confidence interval.

^a^Compared to baseline prevalence.

Children aged <5 years had the highest prevalence of scabies at 36 months (12.5%; adjusted odds ratio [AOR], 33.2; 95% CI, 6.6–603.2; Supplementary Table 1) and the smallest decline in prevalence from baseline at both the 12- and 36-month surveys (Supplementary Table 2). No cases of crusted scabies were diagnosed in the study population at 36 months.

At 36 months, 116 people were diagnosed with impetigo (9.6%; 95% CI, 8.1%–11.4%). The prevalence of impetigo was significantly lower than at baseline (24.7%), with an absolute reduction of 15.2% and relative reduction of 61.3% (95% CI, 38.7%–100%; *P* < .001; Table 2). The highest prevalence of impetigo was among children aged 5–9 years (16.4%; AOR, 8.1; 95% CI, 3.6–21.8; Supplementary Table 1). Impetigo was diagnosed in all 10 study villages, with a range of village-level prevalences of 3.7%–17.6% (Supplementary Table 1).

Scabies was significantly associated with impetigo at the 36-month survey (AOR, 4.7; 95% CI, 2.5–8.5; *P* < .001). However, the population-attributable fraction of impetigo due to scabies declined across the 3 surveys from 41% at baseline to 19% and 13% at 12 and 36 months of follow-up, respectively.

### Prevalence of Scabies and Impetigo in Sentinel Villages at 12 and 36 Months

The absolute difference in the prevalence of scabies between the 12-month and 36-month surveys was +2.4% (95% CI, 0.1–3.9), and the absolute difference in the prevalence of impetigo between the 12-month and 36-month surveys was +3.3% (95% CI, 0.9–6.4) (Table 2). The absolute changes in prevalence between baseline, 12 months, and 36 months were similar across all age ranges (Supplementary Table 2).

After adjustment for age and gender, the prevalence of scabies was significantly lower compared to baseline at both the 12-month (AOR, 0.10; 95% CI, 0.07–0.15) and 36-month (AOR, 0.19; 95% CI, 0.14–0.26) surveys. After adjustment for age, gender, and presence of scabies, the prevalence of impetigo was significantly lower compared to baseline at both the 12-month (AOR, 0.31; 95% CI, 0.23–0.41) and the 36-month (AOR, 0.39; 95% CI, 0.30–0.50) surveys. Overall, the changes in both scabies and impetigo prevalence between 0, 12, and 36 months were similar between our main survey findings and the findings from the subset of villages that were visited more than once (Supplementary Table 3).

## Discussion

In this study, we demonstrate the sustained impact of a single round of ivermectin-based MDA on the prevalence of scabies and impetigo for 3 years after intervention. The relative reduction in scabies and impetigo remained >70% and >60%, respectively, at 3 years compared to baseline, representing an ongoing substantial public health benefit from MDA. While scabies and impetigo both increased between month 12 and month 36, the point estimates and CIs are consistent with relatively small increases over the 2-year period. Our data suggest that while further intervention may be required to sustain these gains, MDA for scabies may not need to be delivered annually to confer a significant reduction in morbidity.

While the province-level prevalence of scabies remained low, there was considerable heterogeneity in prevalence; in 1 sampled village at 36 months, the prevalence had returned to its baseline level. In this village, the prevalence of impetigo remained lower than at baseline and was also lower than that found in several other villages at 36 months. We did not identify any cases of crusted scabies or other possible explanations for the relatively rapid recrudescence in scabies prevalence in this village. District-level prevalence estimates may mask focal disease “hot spots” after MDA. Because we only surveyed 10 villages at the 36-month visit, we cannot exclude the possibility that scabies may have also returned to baseline levels (or greater) in other villages in Choiseul Province. Conversely, apparent hot spots may simply represent stochastic noise arising from sampling. If mechanisms for identifying and confirming such hot spots could be validated, mop-up targeted MDA might become a viable strategy to use in these communities, rather than deploying further rounds of district-level intervention.

The prevalence of impetigo was substantially lower at 36 months than at baseline [13]. Our data confirm that in this setting, scabies is the major driver for impetigo and that treatment of scabies results in marked declines in impetigo prevalence. At 3 years, the population-attributable fraction burden of impetigo had declined markedly, suggesting that additional interventions beyond ivermectin-based MDA may be necessary to further reduce impetigo prevalence. We previously demonstrated that the addition of single-dose azithromycin administered at the same time as ivermectin as part of MDA does not appear to drive greater reductions in impetigo [12]. It is plausible that the remaining impetigo burden is a result of multiple distinct risk factors and mechanisms, such as skin breaches from localized trauma, insect bites, and fungal and other skin conditions. With a sufficiently low prevalence of impetigo, as achieved following ivermectin-based MDA, a case-management strategy could potentially be used to control residual disease.

At both the 12- and 36-month follow-up visits, the smallest relative reductions in scabies and impetigo prevalence were among children aged 0–4 years, the group who received treatment with permethrin rather than ivermectin. The reductions seen in this age group were similar to those seen in the permethrin MDA arm of the Skin Health Intervention Fiji Trial in Fiji [11]. On an individual level, some data suggest that permethrin is equivalent, if not superior, to ivermectin for the treatment of scabies [21–23]. However, in an MDA context, reduced adherence or inadequate application of permethrin compared to directly observed therapy with ivermectin might attenuate its efficacy. Collectively, these data highlight the need for additional strategies to target scabies in this high-risk age group. One option may be to lower the minimum age and/or weight limit for ivermectin administration.

With the World Health Organization having designated scabies as a neglected tropical disease, there is a pressing need for evidence to inform guidelines for control interventions. In our study, reported coverage was very high, above 95% for the first dose and above 80% for the second dose of treatment. The results suggest that this coverage with 2-dose ivermectin-based MDA may have a large and sustained impact on the prevalence of both scabies and impetigo at 3 years. Our study was nonrandomized and performed in a relatively isolated island population. Despite these limitations, it represents a significant contribution to the emerging data on the use of ivermectin-based MDA for the control of scabies, providing further support for adoption of this intervention as the central component of global scabies control efforts.

## Supplementary Data

Supplementary materials are available at *Clinical Infectious Diseases* online. Consisting of data provided by the authors to benefit the reader, the posted materials are not copyedited and are the sole responsibility of the authors, so questions or comments should be addressed to the corresponding author.

ciz444_Suppl_Supplementary_Material

## Notes

***Author contributions.*** All authors contributed substantially to the design of the study. M. M. was the primary coordinator of data collection for the 3-year follow-up survey and analysis and was the primary author of the manuscript. L. R. conducted fieldwork for the baseline and 12-month surveys. O. S. coordinated the fieldwork in the Solomon Islands. J. M. K. and A. C. S. supervised data collection, analysis, and writing and vouched for the integrity and completeness of the data and analyses. All authors contributed to the writing of the manuscript and read and approved the final version.

***Acknowledgments.*** J. M. K., A. C. S., and D. E. were supported by Australian National Health and Medical Research Council fellowships. A. C. S. is also supported by the National Heart Foundation of Australia. M. M. was supported by the Wellcome Trust (102807) and the UK National Institute of Health Research. A. W. S. was supported by the Wellcome Trust (098521).

***Disclaimer.*** The authors alone are responsible for the views expressed in this article and they do not necessarily represent the views, decisions, or policies of the institutions with which they are affiliated. The funders had no role in study design, data collection and analysis, the decision to publish, or preparation of the manuscript. The corresponding author had full access to all the data in the study and had final responsibility for the decision to submit for publication.

***Financial support.*** The study was funded by the International Trachoma Initiative; the Murdoch Children’s Research Institute, Australia; the Scobie and Claire Mackinnon Trust, Australia; and the Wellcome Trust. Ivermectin was provided at a reduced cost by Merck Sharp & Dohme Australia. Azithromycin was provided directly to the Ministry of Health and Medical Services National Trachoma Control Programme by the International Trachoma Initiative. The Solomon Islands Ministry of Health and Medical Services provided paid personnel and office space.

***Potential conflicts of interest.*** All authors: No reported conflicts of interest. All authors have submitted the ICMJE Form for Disclosure of Potential Conflicts of Interest. Conflicts that the editors consider relevant to the content of the manuscript have been disclosed.
