# Supplementary material for: Prevalence of Scabies and Impetigo 3 Years After Mass Drug Administration With Ivermectin and Azithromycin
Source: Clin Infect Dis. 2019 May 25;70(8):1591–5. doi: 10.1093/cid/ciz444 (PMC7145994; doi:10.1093/cid/ciz444)
Supplement: ciz444_Suppl_Supplementary_Material [file ciz444_suppl_supplementary_material.docx]

**Supplementary Table 1**

|  |  | **Sample** | **Participants with scabies** | | | **Adjusted OR (95% CI)** | | **Participants with impetigo** | | | **Adjusted OR (95% CI)** |
| --- | --- | --- | --- | --- | --- | --- | --- | --- | --- | --- | --- |
|  |  | **n** | **n** | **%** | **95% CI** |  | **n** | | **%** | **95% CI** |  |
| **Total** |  | 1,210 | 57 | 4.7 | 3.6-6.1 |  | **116** | | **9.60%** | 8.1 – 11.4 |  |
| **Gender** | Female | 664 | 27 | 4.1 | 2.7 – 5.9 | 1 (ref) | **58** | | 8.7 | 6.7 – 11.2 | 1 (ref) |
|  | Male | 546 | 30 | 5.5 | 3.8 – 7.8 | 1.3 (0.7 – 2.3) | **61** | | 11.1 | 8.7 – 14.2 | 1.1 (0.7 – 1.6) |
| **Age (years)** | <5 | 160 | 20 | 12.5 | 7.9 – 18.9 | 33.2 (6.6 – 603.2) | **22** | | 13.8 | 9.0 – 20.3 | 7.6 (3.1 – 21.2)) |
|  | 5-9 | 269 | 14 | 5.2 | 3.0 – 8.8 | 10.4 (2.0- 190.9) | **44** | | 16.4 | 12.3 – 21.4 | 8.1 (3.6- 21.8) |
|  | 10-14 | 277 | 13 | 4.7 | 2.6 – 8.1 | 12.6 (2.4 – 231.1) | **30** | | 10.8 | 7.5 – 15.2 | 4.6 (2.0 – 12.5) |
|  | 15-24 | 121 | 4 | 3.2 | 1.0 – 8.5 | 6.9 (1.0 – 138.3) | **12** | | 9.9 | 5.5 – 17.0 | 3.4 (1.2 – 10.2) |
|  | 25-34 | 118 | 5 | 4.2 | 1.6 – 10.1 | 11.8 (1.8 – 230.5) | **5** | | 4.2 | 1.5 – 10.1 | 1.7 (0.5 – 5.9) |
|  | ≥35 | 265 | 1 | 0.4 | 0.0 – 2.4 | 1 (ref) | **6** | | 2.3 | 0.1 – 5.1 | 1 (ref) |
| **Village** | 21 | 166 | 2 | 1.2 | 0.2 – 4.7 | 0.2 (0.0 – 0.7) | **14** | | 8.4 | 4.8 – 14.1 | 1.3 (0.5 – 4.1) |
|  | 22 | 107 | 5 | 4.7 | 1.7 – 11.1 | 0.8 (0.2 – 2.5) | **4** | | 3.7 | 1.2 – 9.9 | 0.5 (0.1 – 2.1) |
|  | 23 | 82 | 4 | 4.9 | 1.6 – 12.7 | 0.9 (0.2 – 2.8) | **6** | | 7.3 | 3.0 – 15.8 | 1.1 (0.3 – 4.0) |
|  | 24 | 138 | 5 | 3.6 | 1.3 – 8.7 | 0.6 (0.2 – 1.9) | **24** | | 17.4 | 11.7 – 25.0 | 2.7 (1.0 – 8.3) |
|  | 25 | 131 | 7 | 5.3 | 2.4 – 11.1 | 0.8 (0.3 – 2.5) | **8** | | 6 | 2.9 – 12.1 | 0.9 (0.3 – 3.1) |
|  | 26 | 107 | 20 | 18.7 | 12.1 – 27.6 | 3.2 (1.3 – 8.3) | **10** | | 9.3 | 4.8 – 16.9 | 1.1 (0.4 – 3.7) |
|  | 27 | 74 | 4 | 5.4 | 1.7 – 14.0 | 0.9 (0.2 – 3.1) | **13** | | 17.6 | 10.0 – 28.5 | 3.5 (1.2 – 11.6) |
|  | 28 | 148 | 1 | 0.6 | 0.0 – 4.3 | 0.1 (0.0 – 0.5) | **10** | | 6.8 | 3.5 – 12.4 | 1.0 (0.3 – 3.2) |
|  | 29 | 178 | 9 | 5.1 | 2.5 – 9.7 | 1 (ref) | **25** | | 14 | 0.9 – 20.2 | 2.4 (0.9 – 7.6) |
|  | 30 | 79 | 0 | - | - | - | **5** | | 6.3 | 2.4 – 14.8 | 1 (ref) |

**Supplementary Table 2**

|  | **Baseline** | **12 Months** | | | **36 Months** | | | |
| --- | --- | --- | --- | --- | --- | --- | --- | --- |
|  | **Prevalence (%)** | **Prevalence** | **Absolute Change from Baseline** | **Relative Change from Baseline** | **Prevalence** | **Absolute Change from Baseline** | **Relative Change from Baseline** | **Absolute Change from 12 Months** |
| **SCABIES** |  |  |  |  |  |  |  |  |
| 0-4yrs | 26.0 | 9.7 | 16.2 | 62.7 | 12.5 | 13.5 | 51.93 | +2.8 |
| 5-9yrs | 34.0 | 0.9 | 33.1 | 97.36 | 5.2 | 28.8 | 84.71 | +4.3 |
| 10-14yrs | 24.9 | 0 | 24.9 | 100 | 4.7 | 20.2 | 81.13 | +4.7 |
| 15-24yrs | 6.3 | 2.2 | 4.1 | 65.08 | 3.3 | 30 | 47.62 | +1.1 |
| 24-34yrs | 8.5 | 0.8 | 7.7 | 90.59 | 4.2 | 4.3 | 50.59 | +3.4 |
| ≥ 35yrs | 10.0 | 1.9 | 8.0 | 81 | 0.4 | 9.6 | 96 | -1.5 |
|  |  |  |  |  |  |  |  |  |
| **IMPETIGO** |  |  |  |  |  |  |  |  |
| 0-4yrs | 28.1 | 14.8 | 13.3 | 47.34 | 13.8 | 14.3 | 50.89 | -1.0 |
| 5-9yrs | 46.4 | 12.2 | 34.2 | 73.71 | 16.3 | 30.1 | 64.88 | +4.1 |
| 10-14yrs | 43.1 | 7.2 | 35.9 | 83.3 | 10.8 | 32.3 | 74.95 | +3.7 |
| 15-24yrs | 11.4 | 4.4 | 7.0 | 61.41 | 9.9 | 1.5 | 13.16 | +5.5 |
| 24-34yrs | 8.4 | 1.5 | 6.9 | 82.15 | 4.2 | 4.2 | 50 | +2.7 |
| ≥ 35yrs | 10.0 | 1.0 | 9.0 | 90 | 2.3 | 7.7 | 77 | +1.3 |

**Supplementary Table 3** Prevalence of scabies and impetigo in participants in villages visited at more than 1 visit (CI: confidence interval)

| **Village** |  | **Prevalence at baseline** | **Prevalence at 12 months** | **Prevalence at 36 months** | **Absolute change in prevalence at 12 months** | **Relative change in prevalence at 12 months** | **Absolute change in prevalence at 36 months*** | **Relative change in prevalence at 36 months*** | **Absolute change in prevalence between 12 and 36 months** |
| --- | --- | --- | --- | --- | --- | --- | --- | --- | --- |
|  |  | **(95% CI)**  **(n/N)** | **(95% CI)**  **(n/N)** | **(95% CI)**  **(n/N)** | **(95% CI)** | **(95% CI)** | **(95% CI)** | **(95% CI)** | **(95% CI)** |
| **1** | **Scabies** | **15.7%**  (10.7 – 21.9)  (28/178) | **1.3%**  (0.2 – 4.6)  (2/154) | **1.2%**  (0.2 – 4.7)  (2/166) | **14.4%**  (8.8 - 20.6) | **92%**  (55.6 - 100) | **14.5%**  (8.3-20.7) | **92.4%**  (52.9 – 100) | **0.1%**  (2.6. - + 2.4) |
|  | **Impetigo** | **23.6%**  (17.6 – 30.5)  (42/178) | **6.5%**  (3.2 – 11.6)  (10/154) | **8.4%**  (4.8 – 14.1)  (14/166) | **17.1%**  (9.5 – 24.5) | **73%**  (40.4 - 100) | **15.2%**  (7.0 – 23.2) | **64.4%**  (29.7 – 98.3) | **+1.9%**  (4.4 - +8.3) |
| **2** | **Scabies** | **22.1%**  (14.9 – 30.9)  (25/113) | **2.3%**  (0.6 -8.0)  (1/44) | **4.7%**  (1.7 – 11.1)  (5/107) | **19.8%**  (8.2 – 28.6) | **90%**  (37.1 - 100) | **17.4%**  (7.9 – 27.0) | **78.7%**  (35.7 - 100 ) | **+2.4%**  (5.2 - +10.0) |
|  | **Impetigo** | **22.1%**  (14.9 – 30.9)  (25/113) | **0**  (0/44) | **3.7%**  (1.2 – 9.9)  (4/107) | **22.1%**  (11.7 – 30.6) | **100%** | **18.4%**  (9.0 – 27.8) | **83.3%**  (40.7 - 100) | **+3.7%**  (14.6 - +8.9) |
| **3** | **Scabies** | **17.3%** (11.8 – 24.0)  (28/162) | **N/A** | **0.6%**  (0.0 – 4.3)  (1/148) | **N/A** | **N/A** | **16.7%**  (10.0 – 23.2) | **96.5%**  (57.1 - 100) | **N/A** |
|  | **Impetigo** | **30.2%**  (23.8 – 37.9)  (49/162) | **N/A** | **6.8%**  (3.5 – 12.4)  (10/148) | **N/A** | **N/A** | **23.4%**  (14.7 – 32.2) | **77.5%**  (48.7 - 100) | **N/A** |
| **4** | **Scabies** | **18.1%**  (13.9 – 22.9)  (52/299) | **N/A** | **4.9%**  (1.6- 12.7)  (4/82) | **N/A** | **N/A** | **13.2%**  (5.4 – 19.6) | **72.9%**  (29.8 - 100) | **N/A** |
|  | **Impetigo** | **26.8%**  (21.8 – 32.2)  (80/299) | **N/A** | **7.3%** (3.0 -15.8)  (6/82) | **N/A** | **N/A** | **19.5%**  (7.3 -26.8) | **72.8%**  (27.2 - 100) | **N/A** |
| **5** | **Scabies** | - | **3.8%**  (1.4 - 8.2)  (6/156) | **5.1%**  (2.5 – 9.7)  (9/178) | **N/A** | **N/A** | **N/A** | **N/A** | **+1.3%**  (3.8 - +6.2) |
|  | **Impetigo** | - | **0.6%**  (0.0 -3.5)  (1/156) | **14.0%**  (0.9 – 20.2)  (25/178) | **N/A** | **N/A** | **N/A** | **N/A** | **+13.4%**  (7.5 – +19.3) |
| **6** | **Scabies** | - | **7.1%**  (2.4 -15.9)  (5/70) | **5.3%**  (2.4 – 11.1)  (7/131) | **N/A** | **N/A** | **N/A** | **N/A** | **1.8%**  (10.1 - +6.5) |
|  | **Impetigo** | - | **11.4%**  (5.1 – 21.3)  (8/70) | **6.0%**  (2.9 -12.1)  (8/131) | **N/A** | **N/A** | **N/A** | **N/A** | **5.4%**  (14.9 - +4.3) |

* Compared to baseline prevalence
